# Medical Opinion and Movement

**Published:** 1907-09-28

**Authors:** 


### Medical Opinion and Movement.

Following the teaching of Cohnheim it has been generally held that the finer branches of the coronary arteries are "end arteries," and do not anastomose, so that blocking of the main stem would deprive a certain area of blood supply. This appears, however, not to be the case. Early this year Janim and Merkel published a series of stereoscopic Röntgen photographs of specially injected hearts, showing the presence of numerous anastomoses between the finer branches of the right and left coronary arteries. More recently Professor Spalteholz has devised a new injecting medium by means of which, together with a process for making the injected hearts transparent, he has succeeded in producing specimens which clearly prove that the coronary arteries are not "end arteries" in Cohnheim's sense. The specimens were exhibited at the last meeting of the German Anatomical Society at Würzburg. They show free anastomoses on the superficial surface, in the substance of the muscle-wall, under the endocardium and in the papillary muscles.

There has been quite an epidemic of small-pox in Vienna, and a consequent rush to the public vaccination stations and to medical men for vaccination or re-vaccination. From 25,000 to 30,000 people have been vaccinated every day. The facts in regard to the incidence of the disease and the mortality in relation to vaccination are interesting, and should afford food for reflection by the anti-vaccinators, had they but eyes to see and ears to hear. Seventy-six per cent. of those attacked have either not been vaccinated at all or have not been re-vaccinated within the last 20 years, whilst the remainder are said to come from the district where the disease was prevalent, and had been infected before the protective influence of re-vaccination had time to be developed. The mortality amounts to 10 per cent. of the persons attacked, but of those who have succumbed none had ever been vaccinated. Such facts and figures are, of course, but a repetition of previous records, and should be sufficiently convincing to any individual of average intelligence. In spite of mischievous endeavours of "nature-cure" partisans to shake the public confidence in the beneficial effect of vaccination, it is said that the attitude of the general population has been quite praiseworthy.

At a recent inquest Mr. Troutbeck, Coroner for Westminster, voiced the complaint that has often been uttered before, that many inquests are held in cases in which any such procedure is quite unnecessary. Under the existing law the coroner is bound to hold an inquest under certain clearly defined circumstances—when he "is informed that the dead body of a person is lying in his jurisdiction and there is reasonable cause to suspect that such person has died either a violent or an unnatural death, or has died a sudden death of which the cause is unknown." On the other hand, it is left largely to the discretion of the coroner to decide whether a post-mortem examination shall be made in any particular case. The contention is that the coroner should have power to order a post-mortem examination in the first place, and then decide according to the results of such an examination whether an inquest is necessary in accordance with the Act. If the post-mortem, for instance, revealed the fact that a sudden death had ensued from a natural cause, such as cardiac disease, there would be no necessity to hold an inquest. Under existing conditions it not infrequently happens that no post-mortem examination is made in cases in which the cause of death is only surmised, but not definitely known, and the inquest does not carry one a step farther towards the truth. One point is clear, however, that if further discretionary powers were given to coroners in the direction indicated, it would become a more urgent necessity that they should possess both legal and medical qualifications. If a coroner is to decide upon the holding of an inquest from the consideration of medical evidence a proper medical training would be an essential qualification.

Gastrojejunostomy is an operation which has found such favour among surgeons in recent years in the treatment of intractable gastric ulcers and other allied conditions, that it is of considerable importance to determine how far the operation affects the normal physiological processes of digestion and metabolism of the body. Mr. Hubert J. Paterson, surgeon to the London Temperance Hospital, has made a special study of the subject, and the results of his observations are decidedly reassuring. In the first place, he finds that the regurgitation of bile into the stomach diminishes in amount in the course of a few weeks, and in any case does not interfere with the digestion or general health. On the contrary, he contends that the beneficial effect of the operation on gastric ulcer is, in part at least, due to a neutralisation of the acid contents of the stomach by the alkaline pancreatic juice and bile. A diminution of the total acidity of the gastric contents is the most marked physiological effect of the operation, and he attributes this to the cause just referred to and also to a diminution of the total chlorides. The motility of the stomach, if normal before operation, is practically unaffected. Mr. Paterson, therefore, discards the view that gastrojejunostomy is a drainage operation. He attributes its virtue rather to the diminution in gastric acidity. His observations on general metabolism are highly interesting. These were carried out on nine different patients who had undergone the operation at different intervals of time. Four of these were placed on a mixed diet and five on a milk diet. The amount of nitrogen and fat absorbed from the food were estimated. Taking the figures of average absorption of nitrogen and fat given by Harley and Goodbody for healthy individuals, namely, 93.46 per cent. nitrogen and 95.05 per cent. fat, he finds that in each case the absorption was only slightly below these amounts, the average for the nine cases being, for nitrogen 91.76 per cent. and for fat 93.15. He is, therefore, justified in concluding that gastrojejunostomy has no material effect on the metabolism of the human body.

